# Single and multi-subject clustering of flow cytometry data for cell-type identification and anomaly detection

**DOI:** 10.1186/s12920-016-0201-x

**Published:** 2016-08-10

**Authors:** Maziyar Baran Pouyan, Vasu Jindal, Javad Birjandtalab, Mehrdad Nourani

**Affiliations:** 1 Quality of Life Technology Laboratory, The University of Texas at Dallas, Richardson, Texas USA; 2 Department of Computer Science, The University of Texas at Dallas, RichardsonTexas, USA

**Keywords:** Anomaly detection, Biaxial gating, Cell-type population, Flow cytometry, Single-cell technology, Two-stage clustering

## Abstract

**Background:**

Measurement of various markers of single cells using flow cytometry has several biological applications. These applications include improving our understanding of behavior of cellular systems, identifying rare cell populations and personalized medication. A common critical issue in the existing methods is identification of the number of cellular populations which heavily affects the accuracy of results. Furthermore, anomaly detection is crucial in flow cytometry experiments. In this work, we propose a two-stage clustering technique for cell type identification in single subject flow cytometry data and extend it for anomaly detection among multiple subjects.

**Results:**

Our experimentation on 42 flow cytometry datasets indicates high performance and accurate clustering (F-measure > 91 %) in identifying main cellular populations. Furthermore, our anomaly detection technique evaluated on Acute Myeloid Leukemia dataset results in only <2 % false positives.

## Background

### Motivation

Flow cytometry is a high-throughput, laser-based technology to study cellular heterogeneous populations [1]. It has revolutionized clinical immunology and healthcare research by providing single-cell level quantifications of various heterogeneous cellular markers (e.g. proteins). These single-cell measurements provide vital insights in correlating phenotypic properties with heterogeneity. Additionally, single cell analysis helps in identification of biomarkers for functional classification and is vital in providing information about the core behavior of complex cellular systems like cancerous tissues. In recent years, there has been a widespread interest in development of flow cytometry tools. The original flow cytometry tools were only able to capture measurements of a single fluorophore. However, current fluorescence based flow cytometers can simultaneously extract measurements of up to 20 cellular markers [2].

Analysis of the flow cytometry data is considered to be one of the most challenging and time-consuming steps in flow cytometry experiments. This is primarily due to the absence of an efficient automatic analysis approach to analyze the high dimensional data generated by advanced flow cytometers. Thus, there is high demand for bioinformatics tools for automatic analysis of flow cytometry data.

Flow cytometry data analysis includes a crucial step called gating which refers to the identification of homogeneous populations of cells with a common specific function. This identification of cell subtypes can be viewed as an unsupervised clustering problem. Gating has traditionally been performed as a manual process. A gate is a defined region of measurement of two cell markers. In manual gating, cells assigned to one gate are visualized in a biaxial plot. In recent years, tools such as FlowJo [3] and FlowCore [4] have enabled researchers to view flow cytometry data as biaxial plots of two parameters. A major drawback in manual gating is the requirement of the user to manually draw gates. The user selects the particular phenotypes on the biaxial plots based on prior experience and intuitive interpretation of density contour lines. Thus, manual gating has been largely criticized for being error-prone due to inter-operator variability, highly subjective and labor intensive. Manual gating, in general, is not a very reliable and efficient to analyze flow cytometry data.

Detection of outliers and anomalous behavior is a well-known problem in the field of data mining. Although the problem of identifying outlier instances (e.g. anomalous cells) within one single subject dataset has been studied in the literature, little effort has been made on detection of anomalous datasets among multiple subject’s datasets. These rare datasets can be similar or dissimilar to each other but can be significantly different from other datasets. For example, in flow cytometry data analysis we obtain multiple datasets and aim to identify datasets with significant differences from others. This identification is very valuable in unsupervised analysis of measured flow cytometry data from different subjects who are treated with one common medicine. During treatment, there may be some subjects with very abnormal response (either positive or negative) to a particular medicine compared to other subjects. This anomalous behavior can occur due to various biological factors. Therefore, identification of abnormal datasets can provide insights to investigate the biological factors responsible for such anomalous behavior.

### Prior works

Numerous comprehensive techniques have been proposed to automate gating in flow cytometry. Primary works in this field automatically extracted cellular populations using regression and classification approaches [5]. However, many of these techniques were inefficient, mainly due to the unavailability of large training datasets. Unsupervised machine learning methods, such as K-means clustering and Gaussian mixture modeling were also utilized to identify clusters from flow cytometry data [6]. Nevertheless, these approaches lack robustness as they are highly sensitive to cluster centers and shapes. Furthermore, these clustering techniques require advance knowledge of number of clusters which is unknown due to heterogeneous cell populations. Methods like Gaussian Mixture Model also assume that each component follows Gaussian distribution which may not always be true in single-cell cytometry data.

A method based on pairwise comparisons and Pearson coefficients is presented in [7]. However, this approach requires a huge computation time as it requires a pairwise distance matrix of order *n*^2^, where *n* is the number of cells. flowPeaks, an approach based on spatial exploration of histograms and finite mixture model is presented in [8]. Despite its computational time efficiency, flowPeaks suffers from a drop in accuracy with an increase in the number of markers. Authors in [9] apply a cluster-merging algorithm on a mixture of t-distributions to enable the model to fit concave cell populations. This method uses Bayesian Information Criterion (BIC) to estimate the number of populations. Nevertheless, BIC may cause extraction of numerous redundant populations. Another method called *FlowMeans* is presented in [10]. *FlowMeans* estimates the maximum number of initial clusters and subsequently merges them together on the basis of their corresponding Mahalanobis distance [11]. The method employs a change point detection algorithm to determine the number of subtypes. However, FlowMeans has a severe limitation in cases with non-existence of the covariance matrix resulting in an undefined Mahalanobis distance. Mathematically, the covariance matrix does not exist when the data has higher dimensions than the number of data points in the cluster. This situation may arise in flow cytometry data set when there are some small populations primarily, due to noise and/or rare subtypes. Another technique using spectral clustering is employed in [12] to extract cellular clusters. Spectral clustering uses an applied sampling procedure which reduce the quality of the results due to loss of critical biological information.

Authors in [13], propose a finite mixture modeling approach called FLAME to automate multivariate estimation. FLAME uses a skew t-distribution mixture model to cluster fluorescence intensity matrices where rows are cells and columns are antibodies. Spanning-Tree Progression Analysis of Density Events (SPADE) method is proposed in [14] to define cellular populations and extract an underlying phenotypic hierarchy tree structure. Although SPADE is an effective technique to visualize high dimensional flow cytometry data, it requires the user to pre-specify the number of initial clusters to extract Minimum Spanning Tree (MST). For example, user can set 100 clusters for a dataset with 8 cellular markers [14]. Furthermore, an increase in number of cellular markers results in an increase in the number of required clusters to extract using SPADE. This creates a bias problem regarding a quantity that is rarely known (number of populations). The user is also required to manually select cellular populations from the produced tree-like structure.

Regarding anomaly detection in flow cytometry, authors in [15] propose an automatic technique to identify rare cell populations in dataset from mice with Acute Myeloid Leukemia (AML). A robust technique based on modified Support Vector Machine (SVM) is presented in [16] to identifying rare cells within a single flow cytometry dataset. Furthermore, when analyzing multiple datasets, Bayesian approach has been proposed to identify rare cell types that are common among all datasets [17]. Authors in [18], discuss several applications of detecting rare events in flow cytometry analysis.

### Main contribution

In this paper, a clustering technique is proposed as the basis for: (i) cell-subtype identification for one subject dataset, and (ii) anomaly detection within datasets of multiple subjects. Our key contribution in this paper is two-fold. First, we propose an approach to identify homogeneous cell subtypes from a single subject flow cytometry dataset. We use a Fuzzy-C-Means and Markov clustering based technique and evaluate our method using three public-domain flow cytometry benchmarks. Second, we extend the approach for anomaly detection, in which datasets from multiple subjects are simultaneously analyzed and anomalous datasets will be identified. To the best of our knowledge, this is the first work proposed to identify anomalous datasets within multiple flow cytometry datasets.

The rest of the paper is organized as follows. We first present our initial work [19] to identify cell types within single subject dataset. Then, in the next section we focus on anomaly detection approach among multiple subject datasets. Subsequently, we discuss our experimental results in the experimental section. Finally, the last section summarizes the paper and presents conclusions and future work.

## Method

### Cell-type identification methodology

**Fig. 1:**
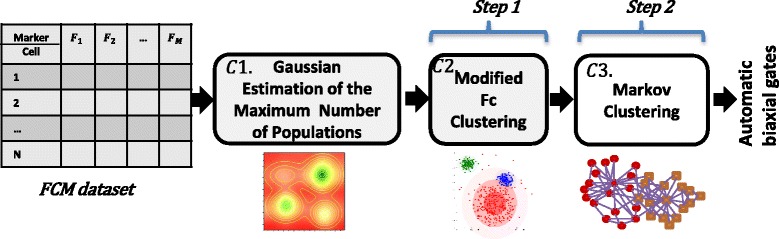
Cell-type identification

Assume that we have a high-dimensional flow cytometry dataset. This dataset includes *N* cells, *X*=[*x*_1_,*x*_2_,…,*x*_*N*_]^⊤^, such that each cell *x* has M cellular markers *x*_*i*_=(*x*_*i*1_,*x*_*i*2_,…,*x*_*iM*_). The goal is to identify homogeneous populations from the data. At first, a reasonable maximum number of populations is estimated. Afterward, a revised version of *Fuzzy-C-Mean* clustering [20] is applied to identify initial clusters from the data. Markov clustering (MCL) [21] is applied on the cluster centers to automatically capture distinct number of populations. Next, the most correlated initial clusters are merged together to find the final cellular populations. Finally, the computed labels are assigned to the cells in each biaxial plot to visualize the extracted subtypes. Figure 1 shows an overall view of our work. Each step of the proposed method will be discussed in detail in next subsections.

### Estimation of initial number of clusters

We estimate the initial number of clusters based on an appropriate maximum number of populations. Maximum number of populations can be estimated by computing the total number of modes found in all eigenvectors of the data [10, 22]. Modes in each eigenvector of the data are detected using kernel density estimation. Then, significance test of the gradient and second derivative of a kernel density estimation is computed according to method described in [23]. Briefly, if *E*={*e*_1_,*e*_2_,…,*e*_*M*_} denotes eigenvectors of dataset *X*, a kernel Gaussian is considered as follows:

1 $$  \kappa(l)=\frac{1}{\sqrt{2 \pi}}exp \left(\frac{-l^{2}}{2}\right)  $$

The kernel density estimator $\hat {f}$ is considered as a mean of *N* Gaussian kernel estimation:

2 $$  \hat{f}(l)=\frac{\sum_{i=1}^{N} \kappa\left(\frac{l-e_{i}}{h}\right)}{N\cdot h}  $$

where *κ*(.) is the Gaussian kernel and *h* is the bandwidth defined based on Scott’s rule [24] as follows:

3 $$  h=\frac{7}{2}\cdot \sigma^{*}\cdot N^{\frac{-1}{3}}  $$

where *σ*^∗^ is the standard deviation of *e*_*i*_ [13].

The estimator gradient is written as follows:

4 $$  \Delta \hat{f}(l)=\frac{2}{N\cdot h^{2}}\cdot \sum\limits_{i=1}^{N} \kappa\left(\frac{l-e_{i}}{h}\right)\cdot(l-e_{i})  $$

Afterward, a simultaneous significance test, using Bonferroni’s correction [25], is applied to find where the gradient is notably different from zero [23]. The number of modes is approximated using the number of times, the gradient changes from positive to negative for each projection of data on the eigenvectors. *K* represents initial number of clusters approximated by summation of all the modes in eigenvectors.

### Initial clustering using Fuzzy-C-Mean

Clustering is an unsupervised learning technique since it categorizes unlabeled instances into meaningful groups using their similar properties. The slight variation of cellular phenotypes are used to probabilistically find different types of cells among flow cytometry dataset. Accordingly, an improved version of Fuzzy-C-Means clustering is developed to calculate the membership probability of each cell when it presumably belongs to a cell population. Fuzzy-C-Means is a soft clustering method which is faster than GMM. Concisely, *χ*={*μ*_1_,…,*μ*_*j*_,…,*μ*_*K*_} will be centers of *K* cellular population *C*={*c*_1_,…,*c*_*j*_,…,*c*_*K*_} which represents potential similarities of M-dimensional cells *X*=[*x*_1_,*x*_2_,…,*x*_*n*_]^⊤^. Cells are assigned to different cell populations (clusters) by minimizing the following optimization model:

5 $$ \left\{ \begin{array}{ll} Minimize~ \left\{J_{m}=\sum_{i=1}^{N}{\sum_{j=1}^{K}{u_{ij}^{m} D_{m}(x_{i},\mu_{j})}}\right\}\\ \text{subject to}: ~\sum_{j=1}^{K}{u_{ij}=1~~~~~~~~\forall i=1,2, \ldots, N } \end{array} \right.  $$

where cell *x*_*i*_ belongs to population *c*_*j*_ with the membership probability of *u*_*ij*_. Fuzzification coefficient is selected as *m*=2 in this work which is empirically reported as *m*≥1 in literature. *D*_*m*_(*x*_*i*_,*μ*_*j*_) implies the *Mahalanobis Distance* between cell *x*_*i*_ and population *c*_*j*_. Note that the shorter distance between cell *x*_*i*_ and the center of population *c*_*j*_, the higher is the probability of *x*_*i*_ belonging to the population *c*_*j*_. Since membership probability depends on the dispersion of population *c*_*j*_, we use Mahalanobis Distance instead of Euclidean Distance as a distance metric between cell *x*_*i*_ and population *c*_*j*_. Let *s*_*j*_ denotes the *M*×*M* covariance matrix of population *c*_*j*_ indicating the direction in which population *c*_*j*_ is spread. The Mahalanobis Distance is represented by the following equation:

6 $$  D_{m}\left(x_{i},\mu_{j}\right)= \sqrt{\left(x_{i}-\mu_{j}\right)\cdot s_{j}^{-1}\cdot\left(x_{i}-\mu_{j}\right)^{T}}  $$

A Lagrangian multiplier defined in [20] is used to minimize the optimization problem of Fuzzy-C-Means given in Eq. 5. The result is a double-step iterative solution computing centroid *μ*_*j*_ and probability *u*_*ij*_(∀*i*,*j*:1≤*i*≤*N*,1≤*j*≤*K*), such that:

7 $$ \left\{ \begin{array}{ll} \mu_{j}^{+}=\frac{\sum_{i=1}^{N}{u_{ij}^{m}.x_{i}}}{\sum_{i=1}^{N}{u_{ij}^{m}}} & \forall j \\ u_{ij}^{+}=\frac{1}{\left[\sum_{k=1}^{K}{\left(\frac {||x_{i}-\mu_{j}||}{||x_{i}-\mu_{k}||}\right)}\right]^{2}} & \forall i,j \end{array} \right.  $$

where $\mu _{j}^{+}$ and $u_{ij}^{+}$ indicates the updated values in the next iteration. The initial cluster set *C* will be available after applying the revised Fuzzy-C-Means.

### Merging clusters using Markov clustering

The number of initial populations may have been overestimated by kernel density estimation in the first stage. This implies that there may be extra populations within the obtained clusters due to projection of clusters on more than one eigenvector. Hence, it is critical that after clustering the cells into *K* initial groups, the redundant clusters should be merged. We address this need using Markov clustering, a fast, divisive and scalable clustering algorithm based on stochastic modeling of flow of networks. To do that, we apply Markov clustering on the initial cluster centers *μ*_1_,…,*μ*_*K*_ to extract the main skeleton of the data cloud. Also since Markov clustering groups *c*_*i*_s are based on their natural affinity, it locates the *c*_*i*_s from the same types in a single cluster. This implies that similar initial populations have closer interaction with each other.

Markov clustering (MCL) has recently emerged as a popular clustering technique in bioinformatics domain for determining cluster networks as well as protein-protein interaction (PPI) networks [26, 27]. The algorithm computes the probability of random walks through a graph by applying two main methods: expansion and inflation. Stochastic matrices, also known as Markov matrices are used in this algorithm due to their capability to represent transition probabilities between all pairs of nodes.

Applying MCL on the initial cluster centers *μ*_1_,…,*μ*_*K*_ results in a gradual determination of the underlying structure of the graph. MCL extracts cellular population by identifying convergence regions with strong internal flow separated by boundaries where flow is absent. The algorithm behind MCL is simple yet efficient: subtypes from the same cell contain links with higher weights than the weights between the different subtypes. Consequently, this implies that a random walk that visits a dense cluster has a higher probability to stay in the same cluster until all its edges have been visited rather than including edges outside the cluster. Furthermore, there is a higher probability of random walks with beginning and ending in the same dense cluster.

The new *stochastic matrix* denoted by *S* is obtained after normalizing columns of adjacency matrix of cluster centers denoted by *A*. As mentioned, the MCL algorithm consists of two main steps: (i) *Expansion*: the power of the matrix is calculated in this step, and (ii) *Inflation*: the *element-wise* product of matrix *S* is calculated and the matrix is rescaled to return it to a stochastic state. Practically, expansion reduces the heterogeneity of flows (random walks) by modeling the spreading out of the flow (free flow). On the other hand, the inflation step strengthens flow in the regions with strong flow while reduces flow in the weak flow region. These steps are repeated until the graph is partitioned into subsets and a stable solution is achieved. This implies that there are no longer any links between the isolated subsets. Finally, the normalized adjacency matrix *S* includes the final isolated segments. The final cellular populations can be extracted from the collection of these isolated segments.

**Fig. 2:**
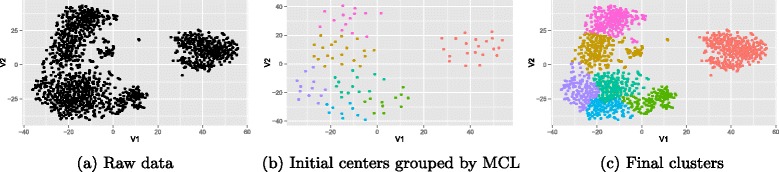
An example of applying our method to a synthesis 2D data

**Fig. 3:**
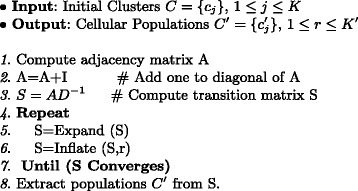
MCL applied to the center of populations

When MCL is applied on centers of initial clusters, the centers corresponding to initial populations will be clustered in the same segments. We extract the final populations by merging these clusters. Figure 2 illustrates an example of applying the proposed combination of applied method on a 2-D simulated data with seven original populations. The pseudo-code of MCL is shown in Fig. 3. *AD*^−1^ denotes normalizing columns of the adjacency matrix *A* so that they sum to one.

## Flow cytometry anomaly detection

In this section, we extend our proposed clustering approach to identify subjects with abnormal cellular behavior using their flow cytometry data. Anomaly detection is different from cell-type identification in terms of input dataset. In cell-type identification, we separately analyze each single subject dataset while anomaly detection holistically investigates all flow cytometry datasets obtained from multiple subjects.

**Fig. 4:**
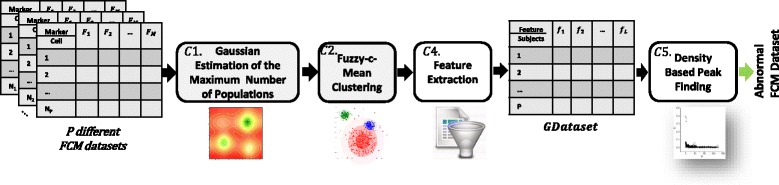
Flow cytometry anomaly detection system

A reliable model is necessary to overcome important challenges in this approach including dependency on subject dataset size and cellular structure. Hence, we employ the proposed cell identification approach (proposed in Section Cell-Type Identification Methodology) with a new density-based anomaly detection technique. Figure 4 illustrates an overview of our proposed method and will be explained in the following subsections.

### Feature extraction

**Fig. 5:**
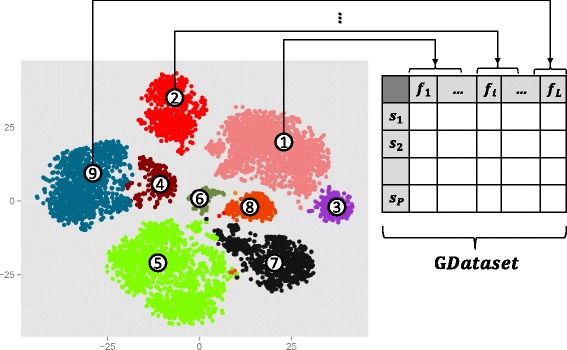
Feature extraction

All datasets corresponding to different subjects are combined to obtain a single big dataset. The two steps (C1 and C2) of cell-type identification stage is applied on this big dataset. Figure 5 symbolically illustrates the process of feature extraction. The feature extraction process is applied for each subject *s*_*i*_ in each cluster to produces a new abstract dataset denoted by *GDataset*. We extract two types of features from each subject in each cluster as follows: (i) the Median Fluorescent Intensity (MFI) [28] of each subject’s protein marker in a particular cluster and (ii) percentage of a subject’s cells accurately assigned to that identified cluster.

### Density-based peak calculation

Let *GDataset*={*s*_1_,*s*_2_,…,*s*_*P*_} denotes the new abstract extracted dataset from *P* subjects. Each element *y*_*i*_ denotes one row of *GDataset* representing subject *i* with *L* related features *y*_*i*_=(*f*_*i*1_,*f*_*i*2_,…,*f*_*iL*_). In this work, we define abnormal (outlier) instances to those instances located in isolated areas and which are far from the normal points in data space of *GDataset*.

Note that anomalous datasets appear individually or as rare subsets of *P*. In this work, we assume the number of instances in rare subsets contain less than 1 *%* of the total number of instances in *GDataset*. We denote this rarity threshold by $\gamma =\lfloor \frac {P}{100}\rfloor $.

In other words, abnormal instances have very low neighborhood density compared to the other data points. We apply an effective density-based peak finding method to identify two critical parameters for each data point.

The peak finding methodology presented in [29] is used to determine all dominant peaks in data. Although this method is designed to identify data clusters, we leverage a new context of this technique to identify outlier data points in *GDataset*. Briefly, the potential high dense data points are assumed to be encircled by other data points with lower local density. Let *ρ*_*i*_ denotes the local density of *s*_*i*_ where *ρ*_*i*_ is the number of *s*_*i*_ that are closer than a predefined cut-off threshold [29]. However, this approach requires to specify a predefined cut-off threshold. As this cut-off threshold is constant for all *s*_*i*_’s, it may cause inaccuracy due to the density variation embedded in each area. To overcome this drawback, we propose to define *ρ*_*i*_ as the mean distance between *s*_*i*_ and its *ω* nearest neighbors:

8 $$  \rho_{i}=\frac{\omega}{\sum_{j=1}^{\omega}||s_{i}-s_{j}||^{2}}~~~~~~1\leq i\leq P  $$

where *ω* is computed as a percentage of the total number of datasets and is defined by:

9 $$ \omega=r\cdot P  $$

Parameter *r* is called density parameter and empirically considered to be *r*=0.15. Our experiments have shown the robustness of our method when *r* is chosen in the range of [0.1, 0.2]. When *ρ* is computed for all the datasets, we put the first *γ* nearest neighbor (based on the *ρ* value) of dataset *i* in set denoted by *ψ*_*i*_. Then, the distance parameter *δ* is computed for each dataset *i* as follows:

10 $$  \delta_{i}=\frac{\sum_{j:j\in \psi_{i}} \{||s_{i}-s_{j}||\}}{N(\psi_{i})}~~~~~~1\leq i\leq P  $$

where ||*s*_*i*_−*s*_*j*_|| denotes the Euclidean distance between dataset *s*_*i*_ and *s*_*j*_. Also, *N*(*ψ*_*i*_) indicates the number of elements in the set *ψ*_*i*_. However, in the case *s*_*i*_ with the highest density denoted *ρ*_*max*_, there will no dataset *i* with density *p*_*i*_ such that *ρ*_*i*_>*ρ*_*max*_. We address this issue by taking $\delta _{i}=\underset {1\leq j\leq P}{\operatorname {max}} \left \{||s_{i}-s_{j}||\right \}$ for *s*_*i*_ with density *ρ*_*max*_.

**Fig. 6:**
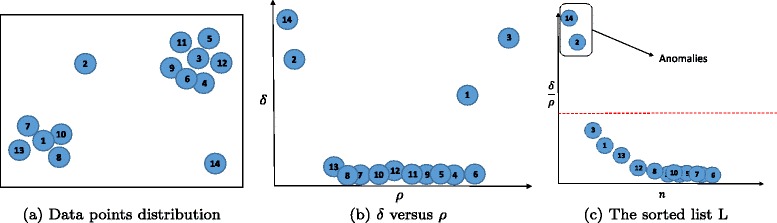
Example of determining data sub-clouds

Let $L=\left \{\frac {\delta _{1}}{\rho _{1}}, \frac {\delta _{2}}{\rho _{2}}, \ldots, \frac {\delta _{K}}{\rho _{K}}\right \}$ and the the anomalous subjects neds to be assigned as outliers (with extremely high values) in this list *L*. These can be easily identified by applying chi-square outlier detection technique [30]. Figure 6 depicts all the mentioned process using an example. According to Fig. 6a, there are two potential clusters. Furthermore, two required parameters *δ* and *ρ* are computed for each data point and then plotted on a 2D map in Fig. 6b. According to Fig. 6b, the dominant peaks (1 and 3) and two anomalous points (2 and 14) have high values of *δ*. Finally, Fig. 6c illustrates the sorted subjects based on the calculated $\frac {\delta }{\rho }$ factor. It is evident that the $\frac {\delta }{\rho }$ value for two anomalous datasets 2 and 14 are much higher compared to the normal datasets.

## Results and discussion

### Cell-type identification

We performed a diverse set of experiments to evaluate the performance of our automatic cell clustering technique:

#### Datasets and methods

We have evaluated our proposed approach with three different benchmarks that are available to download for free through FlowRepository [31] with the following experiment IDs: FR-FCM-ZZYY (DLBCL), FR-FCM-ZZY2 (GvHD), and FR-FCM-ZZYZ (ND).

1. Diffuse Large B-Cell Lymphoma (DLBCL): A famous lymphoma dataset that includes 30 subjects. DLBCL is the most common lymphoma worldwide. It is an aggressive (fast-growing) lymphoma arising in either lymph nodes or outside of the lymphatic system. DLBCL contains several subtypes that affect its prognosis and it spreads in testes, thyroid, skin, breast or brain. The dataset consists of 30 samples with each sample containing 3 cellular features: *CD*3, *CD*5, and *CD*19. The number of cells ranges from 1000 to 20,000 in each sample set. In addition to three main cellular markers, CD3, CD5, and CD19, two size cellular markers *FS* and *SS* are also measured which are not mostly used in cellular analysis.
2. Graft versus Host Disease (GvHD): GvHD is a type of complication arising after an allogeneic hematopoietic stem cell transplant. In this complication, the donated white blood cells (T cells) in the graft initiate an attack on the skin, gut, liver, and other tissues of the recipient. Previously, gene expression patterns have been extracted using microarrays of peripheral blood leukocytes that are responsible for GvHD diagnosis. However, microarray data is inefficient in identifying gene expressions of heterogeneous peripheral blood leukocytes. This is primarily because microarray analysis outputs similar gene expressions even for heterogeneous populations [32]. This shortcoming may lead to loss of critical variations in expressions of individual genes within different cellular populations. The data set includes 12 samples such that each sample includes 12,000 to 30,000 instances. Each cell has 4 main protein markers: *CD*3,*CD*4,*CD*8 and *CD*8.
3. Normal Donors (ND): The dataset includes 30 healthy subjects with 9 main cell markers. In this dataset, investigators examined differences in the responses of various cell types to different stimuli. The time periods were relatively short in this data to prevent change in surface markers. The staining panel contains antibodies to surface markers and intracellular proteins.

Additionally, we compared our method against four well-known algorithms in the field: FLAME [13], SamSpectral [12], flowMerge [9], and flowMeans [10].

#### Performance evaluation

A challenge in evaluation of the datasets is that all the three datasets use distinct reference labels as the ground truth assigned using manual gating and biological analysis in the laboratory [33]. We address this issue using the harmonic mean of *Precision* and *Recall* or *F-measure*. F-measure is defined as follows:

11 $$  F(L,L')=\frac{1}{N}\sum\limits_{l_{i}\in L}|l_{i}|\times \underset{l'_{j}\in L'}{\max}\left\{F\left(l_{i},l'_{j}\right)\right\}  $$

such that:

12 $$ \left\{ \begin{aligned} &F({l}_{i},{l^{\prime}_{j}})=\frac{2\cdot Recall(l_{i},{l^{\prime}_{j}})\times Precision(l_{i},{l^{\prime}_{j}})}{Recall(l_{i},{l^{\prime}_{j}})+Precision(l_{i},{l^{\prime}_{j}})} \\ &Precision(l_{i},{l^{\prime}_{j}})=\frac{n_{ij}}{l^{\prime}_{j}} \\ &Recall(l_{i},{l^{\prime}_{j}})=\frac{n_{ij}}{l_{i}} \end{aligned} \right.  $$

where |*l*_*i*_| is the number of assigned labels by expert in cluster *c*_*i*_, $l^{\prime }_{j}$ is the number of cells clustered in population *c*_*j*_ found by automatic method. Factor *n*_*ij*_ is the number of cells with label *l*_*i*_ assigned to cluster *c*_*j*_.

**Fig. 7:**
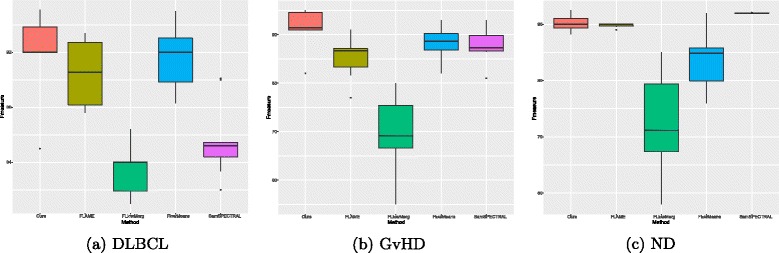
Performance comparison

All experiments were performed using a desktop system with 3 GHz CPU and 8 GB of RAM. Figure 7 shows the calculated F-measure for algorithms applied on three benchmarks. F-measure of each sample was calculated and the average is reported as a single value representative of the F-measure values. For example, the related entry to our method for *ND* represents that the average F-measure for 30 samples is 0.90 in the range of [0.87, 0.92]. According to Fig. 7, in general, our method achieves the best or very comparable results compared to other methods.

Table 1 illustrates the comparison of the computed number of populations using manual analysis and automatic methods. The results show that the number of populations estimated by our method is close to manual analysis results. In particular, for DLBCL, our proposed method estimates an average of 2.5 clusters while other methods estimation is in the range of [2, 5].

**Table 1:** Number of extracted populations

Dataset	Manual	Flow	Flow	Sam	FLAME	Ours
	Gating	Merge	Means	SPECTRAL	[13]	
	[33]	[9]	[10]	[12]		
DLBCL	2 (1–4)	5 (3–8)	3.5 (3–6)	4.5 (2–7)	9 (2–10)	2.5 (2–4)
GvHD	3 (1–5)	6 (3–9)	4 (2–5)	4 (3–7)	5 (1–10)	3.5 (2–4)
ND	6 (3–8)	9 (6–11)	7 (6–13)	10 (5–20)	9 (7–14)	8 (7–12)

Table 2 shows the running time of each method and dataset. Although our method is not as fast as FlowMeans for datasets used in this experiment, FlowMeans is time-consuming when applied on higher dimensional flow cytometry data. This is due to the lengthy nature of the merging step in FlowMeans making it slower than our proposed method.

**Table 2:** Performance comparison (running time in (MM:SS)

Dataset	FlowMerge	FlowMeans	SamSPEC	FLAME	Ours
	[9]	[10]	TRAL [12]	[13]	
DLBCL	11:48	00:26	00:48	00:43	00:29
GvHD	15:41	00:34	01:05	01:23	00:37
ND	23:05	00:46	01:42	01:57	00:58

**Fig. 8:**
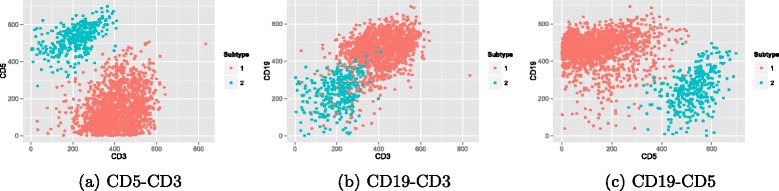
An example of cell-type identification in automatic DLBCL biaxial gating

**Fig. 9:**
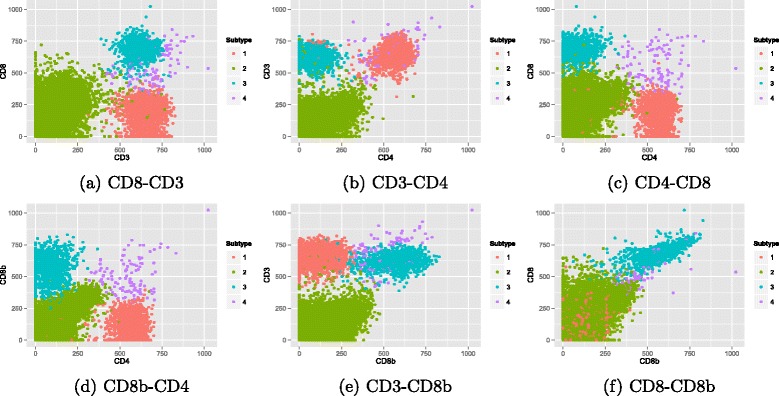
An example of cell-type identification in automatic GvHD biaxial gating

Finally, one sample from each data is selected to automatically visualize the extracted populations on the biaxial plots (Figs. 8 and 9). The comparison of the colorful populations with isolated dense areas in biaxial plots can be used for performance evaluation. In these figures, two extracted populations for DLBCL and four populations for GvHD are visualized in each biaxial gate of related markers.

#### Integration within SPADE

Spanning-Tree Progression Analysis of Density Events (SPADE) is a visualization tool for flow cytometry data. It extracts a hierarchy tree structure from the datasets in an unsupervised manner. Briefly, K-means clustering is used to segment the data into a predefined number of clusters. Then, a Minimum Spanning Tree (MST) is defined on the centers of initial clusters. The tree-like structure is extracted by unfolding the MST on the 2-D space. However, a major drawback of SPADE is that it requires user input to extract cellular populations. Despite of its efficiency in visualizing high dimensional data, SPADE is a parametric technique due to the requirement of initial number of clusters. Therefore, we integrate our approach with SPADE (to be called *AUTO-SPADE*). This integration improves the performance of SPADE which can be used for automated clustering tool without pre-defined number of populations.

**Fig. 10:**
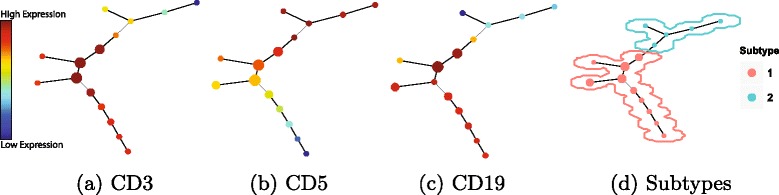
An example of running Auto-SPADE for DLBCL

**Fig. 11:**
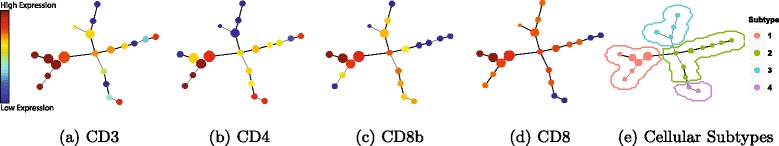
An example of running Auto-SPADE for GvHD

Figures 10 and 11 illustrate the expression value of each protein marker on the tree-structure extracted by AUTO-SPADE for DLBCL and GvHD, respectively. The initial clusters are represented by circles and the red and blue colors denote high and low expression of protein markers, respectively. Figures 10d and 11e visualize the extracted populations on the tree-like structures of SPADE. A large number of clusters is estimated in the beginning by the original SPADE tool. However, according to our method, the initial number of clusters is proportional to the dimensionality of the dataset. For example, the AUTO-SPADE segments DLBCL and GvHD into 15 and 20 initial clusters, respectively. According to Figs. 8 and 9, DLBCL and GvHD datasets were clustered into 2 and 4 populations, respectively.

### Flow cytometry sample anomaly detection

In this section, we evaluate the proposed flow cytometry anomaly detection technique using one publicly available dataset which contains multiple flow cytometry datasets. The raw datasets are freely available through FlowRepository [31] with experiment IDs: FR-FCM-ZZYY. Acute Myeloid Leukemia (AML): The data is collected from 43 subjects with AML positive and 316 healthy donor subjects [33, 34].

We applied our method on AML dataset to validate its ability to distinguish rare flow cytometry datasets corresponding to AML subjects from datasets corresponding to healthy subjects. It is assumed that populations with less than 1 *%* of the total instances are considered as rare instances. In order to show the effective of our anomaly detection approach, we applied our method under four different scenarios:

1. All healthy subjects in conjunction with one AML patient (repeated 43 times for different AML subjects).
2. All healthy subjects in conjunction with two AML patients (repeated 100 times with each time including a random selection of two AML patients out of 43 total AML patients).
3. All healthy subjects in conjunction with three AML patients (repeated 100 times with each time including a a random selection of three AML patients out of total 43).
4. All healthy subjects in conjunction with ten AML subjects (repeated 100 times with each time including random selection of ten AML patients out of total 43).

Table 3 reports the performance of our model in the above four scenarios. For each scenario, the number of false positives and total run time are reported. The total number of false positives in each scenarios is negligible compared to the size of the dataset. Table 3 illustrates that our proposed technique is extremely fast and accurate.

**Table 3:** The performance of the proposed anomaly detection

Experiment	Total number of AML subjects	Number of identified AML subjects	Number of False positives	Runtime (In second)
1	48	47	1	2
2	203	200	3	5
3	902	897	5	12
4	1009	1000	9	19

**Fig. 12:**
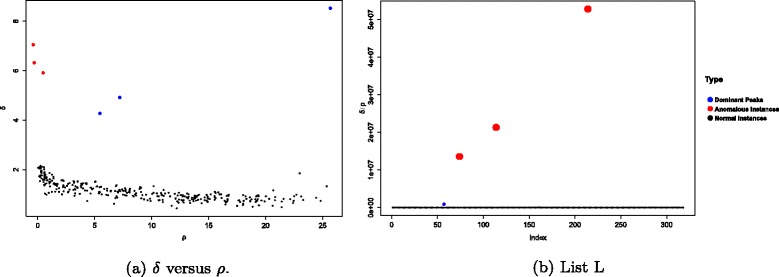
An example of third experiment with three different AML subjects

Figure 12a and b display plot of *δ* versus *ρ* and plot of list *L* respectively for an example case. The identified rare subjects (red points) and dominant peaks (blue points) are well separated and are easily distinguishable in Fig. 12a. Once list *L* is created, the potential rare subjects with large value of $\frac {\delta }{\rho }$ emerged in Fig. 12b.

## Conclusions

In this paper, a novel clustering based approach is presented to identify the main cellular subtypes of multi-variable flow cytometry single subject datasets. We integrated the proposed technique within SPADE analysis tool to automate selection of the number of clusters and extract main cellular populations. Furthermore, we extended this approach to an automatic anomaly detection system to distinguish rare cases in a multi-subject flow cytometry dataset. Our method is fast and can be used to accurately analyze multiple flow cytometry datasets. Our future work includes improving the accuracy of two approaches by applying new distance metric learning. This is expected to improve the performance of our methods to further analyze high-dimensional mass cytometry (CyTOF) datasets.
